# Self‐administered single‐session interventions for mental health in young people: A systematic review and meta‐analysis

**DOI:** 10.1111/jcpp.70192

**Published:** 2026-06-19

**Authors:** Jessica Ball, Richard Meiser‐Stedman, Zoe Thompson, Kenny Chiu

**Affiliations:** ^1^ Department of Clinical Psychology and Psychological Therapies University of East Anglia, Norwich Research Park Norwich Norfolk UK; ^2^ Department of Psychology, Institute of Psychiatry, Psychology & Neuroscience King’s College London London UK

**Keywords:** Anxiety, depression, young people, mental health, self‐administered, single‐session interventions

## Abstract

**Background:**

Children and young people's mental health is an area of concern worldwide. In the face of barriers such as long waits to access evidence‐based treatment, perceived stigma, and a desire to tackle challenges independently, self‐administered single‐session interventions (SSIs) have arisen as one potential solution. The aim of this systematic review and meta‐analysis is to synthesise evidence of the efficacy of self‐administered SSIs for youth mental health.

**Methods:**

This random‐effects meta‐analytic review was preregistered on PROSPERO (CRD42023457030). Systematic search of randomised controlled trials (RCTs) from the Cochrane CENTRAL Register of Trials, EMBASE, MEDLINE and PsychINFO databases was conducted.

**Results:**

Following screening, 25 RCTs met eligibility criteria (5,628 participants), 19 of which were meta‐analysed. Most of the included studies exhibited either ‘Low Risk’ or ‘Some Concerns’, with several of them having a ‘High Risk’ of bias. We found a small effect for anxiety symptoms (*g* = −0.24, 95% CI [−0.41, −0.07]) with substantial heterogeneity (*I* ^2^ = 62.68%) and a wide prediction interval (95% PI [−0.67, 0.19]). Furthermore, there was a small effect for depressive symptoms (*g* = −0.11, 95% CI [−0.22, −0.02]) with substantial heterogeneity (*I* ^2^ = 53.86%) and a wide prediction interval (95% PI [−0.42, 0.19)]. Studies not included in the quantitative synthesis were discussed narratively.

**Conclusions:**

The findings suggest that self‐administered SSIs may be efficacious in reducing anxiety and depressive symptoms in young people. Although the evidence has limitations, including a small number of studies and diverse clinical and statistical characteristics, these findings indicate the need for further exploration of SSIs to address the treatment gap in child and adolescent mental health care.

## Introduction

Increasing timely access to mental health support for young people has become a priority due to the rapidly growing number who experience poor mental health (Edbrooke‐Childs & Deighton, 2020; Racine et al., 2021), especially following the COVID‐19 pandemic (Fegert, Vitiello, Plener, & Clemens, 2020; Racine et al., 2021). However, there are a number of barriers, including long waits and limited‐service provision (Davies, 2023). In addition, patients have identified barriers relating to stigma and discrimination (Tunks, Berry, Strauss, Nyikavaranda, & Ford, 2023). Specifically, young people have reported failing to seek help from professionals due to their preference to deal with difficulties themselves, sense of embarrassment, and poor mental health literacy, as well as fears relating to meeting a new person, lack of time, and transportation barriers (Gulliver, Griffiths, & Christensen, 2010; Radež et al., 2020).

One potential solution to meet public demand is single‐session interventions (SSIs). SSIs are defined as an intervention that intentionally involves only one encounter, on any platform, and can be self‐ (including parent‐ or school‐led) or therapist‐administered, informed by certain theoretical approaches (Schleider & Weisz, 2017). Previously, a review of 50 randomised controlled trials (RCTs) focusing on all SSIs, published by December 2015, found evidence of mental health symptoms relief, with the largest effect sizes seen for the reduction in anxiety symptoms and conduct problems, with no significant statistical differences in efficacy between self‐ and therapist‐administered interventions (Schleider & Weisz, 2017). A more recent meta‐analysis conducted by Wang, Zhang, and An (2023) found that internet‐based self‐help interventions were more effective than control groups, with small‐to‐moderate effects for anxiety and depressive symptoms. A limitation to these reviews is that none of them focused on self‐administered SSIs, which refers to a brief form of intervention that individuals can complete on their own without assistance from others. Self‐administered SSIs have been recognised as important to promote individual's self‐efficacy, motivation, and engagement in their health care (World Health Organisation [WHO], 2022). Being able to self‐administer the interventions means that the intervention can be deployed at scale, where users can access it any time they want.

The increase in RCTs of self‐administered SSIs in recent years (Schleider, Dobias, Sung, & Mullarkey, 2019) provides a unique opportunity to evaluate their efficacy. While systematic reviews focusing on self‐administered SSI have been published using narrative synthesis, none of them employed meta‐analysis to synthesise effect size data (e.g. Lehtimaki, Martic, Wahl, Foster, & Schwalbe, 2021; Wang et al., 2023). A meta‐analysis can be used to aggregate individual RCT effect size data, increasing statistical power to estimate the pooled effect size on each mental health outcome. In addition, meta‐analysis can explore if treatment effect is moderated by factors such as demographic factors, intervention design factors, and type of target problem. Results from these analyses can inform future development and deployment of similar interventions at large scale. As such, this review is designed to systematically review and synthesise current evidence from RCTs of self‐administered SSIs for young people's mental health. SSIs are designed to maximise scalability and reduce barriers to access. However, their effectiveness in practice is shaped not only by intervention content but also by engagement processes. Non‐initiation and early attrition often limiting potential benefits even when interventions are efficacious under optimal conditions. In this review, we discussed these constraints as part of the wider implementation context and considered how engagement challenges may contribute to variability in outcomes across studies.

## Methods

### Study registration

This review is reported following PRISMA guidelines (see Appendix S1 for the checklist), with study protocol registered on PROPSERO (CRD42023457030).

### Eligibility criteria

Eligibility criteria of this review are: (1) must be a randomised controlled trial, (2) must include children and young people under 25 years old, (3) must evaluate a self‐administered SSI, (4) the SSI should aim to improve psychological or emotional outcomes, (5) treatment outcomes must be measured using validated quantitative psychometric measures, (6) must be a peer‐reviewed empirical study published in English. The age range was chosen given research evidencing that the adolescent brain continues developing until 25 years old (Arain et al., 2013).

### Search strategy

The Cochrane CENTRAL Trial Register, EMBASE, MEDLINE, and PsycINFO databases were searched from the first published article to 20 September 2023, using the following search terms: (“single‐session” OR “single session” OR “one session” OR “one‐session” OR “brief intervention”) AND (anxi* OR depress* OR “worry” OR “low mood” OR “mental illness” OR “mental disorder” OR “wellbeing” OR emotion* OR “psychiatric illness” OR phobi* OR panic OR sad* OR shame OR guilt OR “mental health”) AND (adolesce* OR teen* OR student* OR pupil* OR “young adults” OR youth* OR “young people” OR “young person” OR minors OR child* OR paediatric OR paediatric) AND (“RCT” OR randomi?ed OR “controlled” OR “feasibility” OR “pilot”). Reference lists of similar reviews were screened to identify relevant studies. An extended search was run covering articles between 20 September 2020 and 29 July 2024. The same search terms were applied consistently in all the databases.

### Study selection

The first author (JB) conducted title and abstract search and full‐text screening using Rayyan (Ouzzani, Hammady, Fedorowicz, & Elmagarmid, 2016). A random 10% of screened studies at these two stages were independently reviewed by the third author (ZT) to ensure consistency. Discrepancies were discussed between the two raters. There was a 100% agreement rate on all articles reviewed by both.

### Data extraction

The following information was extracted by the first author (JB): author, publication year, country, journal, sample size, sex, ethnicity, primary target problem, intervention and control group type, follow‐up length, intervention and control group length, study type (Targeted treatment [sample is selected based on having a clinical diagnosis], Targeted prevention [selected sample based on risk or an elevated scoring on screening measures] or Universal Prevention [everyone was eligible, regardless of their clinical characteristics or diagnostic status]), mode of delivery, means, and standard deviations (*SD*s) of mental health outcome of interest (where standard error was reported this was converted to *SD*). The third author (ZT) reviewed a random 10% of the extracted data to verify data extraction accuracy. Effect sizes were calculated using the earliest available postintervention mean and *SD* data to reduce variation caused by different follow‐up durations. When both youth‐ and parent‐report data were available (*n* = 2), we reported youth self‐report data to maintain consistency across studies, as this was the most commonly available informant. When a study reported both a broad and a narrow symptom measure, we selected the broad measure (*n* = 1). For example, Schleider et al. (2022) assessed depressive symptoms and hopelessness in their study, and we extracted depressive symptoms to capture the broad depression phenomenology. Schleider et al. (2022) conducted a three‐arm RCT comparing growth mindset intervention, behavioural activation, and control. We selected the results of the growth mindset intervention for the meta‐analysis as this was one of the most studied interventions in this review.

### Risk of bias assessment

Study quality was assessed using the Cochrane Risk‐of‐Bias Tool (RoB‐2; Sterne et al., 2019). The RoB‐2 comprises six domains: selection of reported result, measurement of outcome, missing outcome data, deviations from intended intervention, randomisation process, and overall bias rating. Each domain contains a series of signalling questions to elicit features relevant to risk of bias within each trial. The tool produces an overall risk of bias rating (‘Low Risk’, ‘Some Concerns’, ‘High Risk’). Each included study was evaluated independently by the first author (JB) and the third author (ZT). Where there were discrepancies, an agreement was reached through discussion.

### Data analysis plan

A meta‐analysis was conducted when there were five or more studies with data relating to the same mental health outcome (e.g. anxiety symptoms, depressive symptoms). When there were fewer than five studies on a certain mental health outcome, results were described qualitatively. A negative effect size indicates that participants receiving the SSI scored lower on the mental health outcomes, compared with the control condition. Hedges' *g* was calculated for each study at postintervention and effect sizes were interpreted according to Cohen (2013) whereby 0.2 is considered a small effect, 0.5 a moderate effect, and 0.8 a large effect. Effect sizes were pooled using the metafor package (Viechtbauer, 2010) in R version 4.4.3 (R Core Team, 2025). A random‐effects model was applied as we anticipated a high level of heterogeneity across studies (Dettori, Norvell, & Chapman, 2022). *I* ^2^ statistic was used to assess degree of heterogeneity in effect sizes across studies. Prediction intervals were reported to predict the range in which future studies effect sizes will fall (Borenstein, 2023). Leave‐one‐out analysis was conducted to examine changes in effect sizes when each study was omitted in turn. Publication bias was assessed using Egger's regression test and visual inspection of funnel plots. If asymmetry was detected, the trim‐and‐fill method was used to adjust for possible bias (Duval & Tweedie, 2000). Subgroup analyses were conducted to estimate the pooled effect sizes for studies examining anxiety and depressive symptoms only. Within each symptom domain, further subgroup analyses were conducted to estimate pooled effect sizes for intervention type and control type. Moderator analyses were performed when there are at least 10 studies in the meta‐analysis and each subgroup had at least four observations (Schwarzer, Carpenter, & Rücker, 2015). Meta‐regression analyses were conducted on length of SSI duration and the time to first follow‐up. While the meta‐analyses used unadjusted posttreatment means to facilitate data pooling, the narrative synthesis prioritises reporting of baseline adjusted effects when available to represent treatment efficacy. To enable standardised comparison across the included studies, effect sizes were converted to Hedges' *g* whenever the reported data existed.

## Results

### Study selection and inclusion

The first search yielded 1,550 results after deduplication. A total number of 25 studies was deemed eligible for inclusion (see Figure 1 for study selection procedure). The updated search yielded a further 18 for full‐text screening and three for final inclusion (Gerber, Nahmias, Schleider, & Lerner, 2024; Heaman, Cherry, McMurtry, Giguère, & Lumley, 2023; Steinberg et al., 2024).

**Figure 1:**
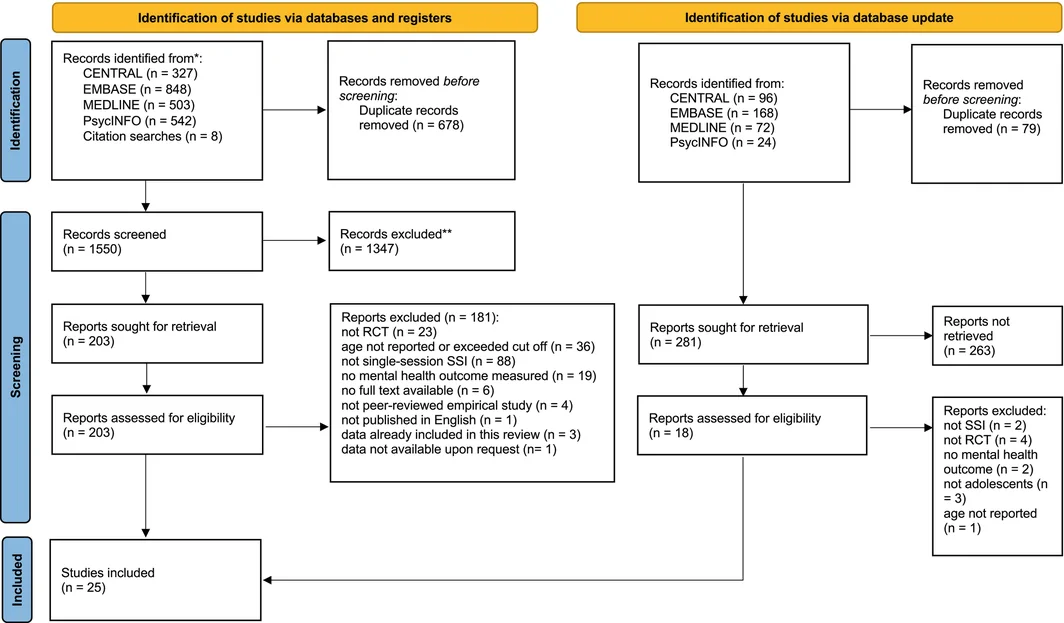
PRISMA diagram

### Characteristics of the included studies

Study characteristics and participant demographic details are summarised in Table 1, and characteristics of SSIs and control conditions are presented in Table 2. Of the 25 studies, 17 included a measure of depressive symptoms, 11 for anxiety symptoms, one explored psychological flexibility, and one respectively for substance use, mental well‐being, and eating disorders. The pooled sample size consists of 5,628 participants, with sample sizes across treatment arms ranging from 13 to 1,631. The ages ranged from 8 to 25 years old. The year of publication ranged from 2005 to 2024. Most of the studies were conducted in the USA, with one study conducted in a lower‐middle income country. The length of the intervention delivery ranged from 1 to 45 min. The dominant gender was female (approximately 70%), and most study participants reported their ethnicity as White.

**Table 1:** Study characteristics

Author (Year)	Target problem (Measure)	Sample size	Age: mean (*SD*)	Ethnicity: % white	Gender: % female	Country
Armitage et al. (2014)	Substance use (TFLB)	67	17.09 (0.38)	90	55.2	UK
Boutelle et al. (2014)	Symptoms of eating disorders (EAH)	29	10.83 (1.28)	55.2	44	USA
Cohen et al. (2023)	Hopelessness (BHS‐4)	54	20.48 (1.81)	NR	46.3	USA
Dobias et al. (2021)	Suicidal ideation (SITBI‐R)	578	14.95 (0.98)	75	66.4	USA
Fitzpatrick, Witte, and Schmidt (2005)	Depressive symptoms (BDI)	110	19.02 (1.21)	75	54.6	USA
Fu, Du, Au, and Lau (2013)	Negative mood (PANAS‐CN)	28	14.5 (1.75)	NR	53.6	China
Fu, Du, Au, and Lau (2015)	Negative mood (PANAS‐CN)	73	14.06 (1.61)	NR	49	China
Gerber et al. (2024)	Depressive symptoms (CDI)	40	11.99 (NR)	75	20	USA
Heaman et al. (2023)	Depressive symptoms (BDI)	312	14.89 (1.54)	60.7	57.9	Canada
Howard, Griffiths, McKetin, and Ma (2018)	Depressive symptoms (SSDS)	236	NR	NR	47	Australia
Norr, Gibby, and Schmidt (2017)	Anxiety symptoms (ASI‐3)	54	19.09 (1.59)	80.0	85.5	USA
Osborn et al. (2020)	Anxiety and depressive symptoms (GAD‐7; PHQ‐8)	103	15.54 (1.22)	NR	64.1	Kenya
Papini, Jacquart, Zaizar, Telch, and Smits (2023)	Anxiety sensitivity (SSASI)	159	19.27 (1.42)	58.4	73.6	USA
Perkins et al. (2021)	Anxiety and depressive symptoms (RCADS‐25); Psychological flexibility (AFQ‐Y8)	80	16.63 (0.56)	81	84	UK
Ranney et al. (2017)	Depressive symptoms (CESD‐10)	357	16.8 (1.3)	57	57	USA
Rheingold et al. (2013)	Anxiety symptoms (STAI‐C)	13	NR	40.6	63.8	USA
Riddleston et al. (2023)	Loneliness (UCLA‐LS)	56	21.57 (1.97)	30.4	83.9	UK
Schleider and Weisz (2016)	Perceived control (PCSC)	96	13.32 (1.14)	73	55	USA
Schleider and Weisz (2018)	Anxiety and depressive symptoms (SCARED‐C; CDI)	96	13.32 (1.14)	73	55	USA
Schleider, Burnette, Widman, Hoyt, and Prinstein (2020)	Depressive symptoms (SMFQ)	211	15.2 (0.5)	38	100	USA
Schleider et al. (2022)	Anxiety and depressive symptoms (GAD‐7; CDI‐SF)	1,631	NR	66.6	88.1	USA
Shapiro, Allan, Raines, and Schmidt (2022)	Anxiety and depressive symptoms (BAI; BDI‐2)	54	18.74 (1.02)	82.1	91.7	USA
Shen et al. (2023)	Anxiety and depressive symptoms (GAD‐7; CDI)	538	15.06 (0.97)	48.1	36.6%	USA
Short and Schmidt (2020)	Anxiety symptoms (BAI)	56	19.43 (2.04)	NR	NR	USA
Steinberg et al. (2024)	Internalising symptoms (BFS)	597	11.99 (NR)	68	48	USA

Schleider and Weisz (2016) and Schleider and Weisz (2018) reported findings based on the same study, but at different follow‐up points. They reported perceived control in 2016, and in 2018 they presented data from a follow‐up whereby anxiety and depressive symptoms were both then reported as outcome measures; therefore, they have been included in the summary table twice even though the sample is the same. ASI‐3, Anxiety Sensitivity Index 3; BAI, Beck Anxiety Inventory; BDI, Beck Depression Inventory; BDI‐2, Beck Depression Inventory‐II; BFS, The Internalising Subscale of the Behaviour and Feelings Survey; BHS‐4, Beck Hopelessness Scale 4 Item; CDI, Children's Depression Inventory; CDI‐SF, Children's Depression Inventory Short Form; CESD‐10, Centre of Epidemiologic Studies Depression Scale, 10‐item version; EAH, Eating in the absence of hunger; GAD‐7, Generalised Anxiety Disorder Screener–7; NR, Not Reported; PANAS‐CN, Positive and Negative Affect Scales for children negative scale; PCSC, Perceived Control Scale for Children; PHQ‐8, Patient Health Questionnaire– 8; RCADS‐25, Revised Child Anxiety & depression Scale, 25 item; SCARED‐C, Screen for Child Anxiety and Related Disorders‐Child version; SITBI‐R, Self‐Injurious Thoughts and Behaviours; SMFQ, Short Mood and Feelings Questionnaire; SPIN, Social Phobia Inventory; SSASI, Short Scale Anxiety Sensitivity Index; SSDS, Self‐Stigma for Depression Scale; STAI‐C, State Trait Anxiety Inventory for Children; TFLB, The timeline follow back; UCLA‐LS, UCLA Loneliness Scale.

**Table 2:** Intervention characteristics

Study	Intervention	Control (active/passive)	Study type	SSI length (min)	Control length (min)	Follow‐up length (weeks)
Armitage et al. (2014)	Self‐affirming	Control placebo (active)	Tar Prev	5	NR	8
Boutelle et al. (2014)	Attention modification	Attention control (active)	Tar Tx	10	NR	0
Cohen et al. (2023)	Enhanced crisis response	Control (active)	Tar Prev	1	1	0
Dobias et al. (2021)	Project Save P‐E	Supportive Therapy (active)	Tar Tx	30	30	12
Fitzpatrick et al. (2005)	P‐E	General health video (active)	Tar Tx	35	35	0, 1, 2, 3
Fu et al. (2013)	CBM‐I	Neutral CBM‐I (active)	Tar Tx	10	NR	0
Fu et al. (2015)	CBM‐I	Control CBM‐I (active)	Tar Prev	10	NR	0
Gerber et al. (2024)	Project Personality	Control (active)	Tar Prev	30	30	0, 13
Heaman et al. (2023)	Growth mindset of personality	Growth mindset of athletic ability (active)	Uni Prev	30	30	0, 17
Howard et al. (2018)	Biological focus P‐E	Control (active)	Uni Prev	40	40	0
Norr et al. (2017)	CAST P‐E	Physical Health Education Training (active)	Tar Prev	35	NR	0
Osborn et al. (2020)	Shamiri‐Digital	Study‐skills control (active)	Uni Prev	60	NR	2
Papini et al. (2023)	CBT based P‐E	Waitlist control (passive)	Tar Tx	60	NR	2
Perkins et al. (2021)	Growth mindset	Waitlist control (passive)	Uni Prev	30	0	0, 4, 8
Ranney et al. (2017)	SafER Teens MI	Enhanced usual care (passive)	Tar Prev	30–45	NR	13, 26, 52
Rheingold et al. (2013)	Video P‐E	Standard care (passive)	Tar Prev	20	N/A	0, 6
Riddleston et al. (2023)	CBM‐I	Control (active)	Tar Prev	NR	NR	NR
Schleider and Weisz (2016)	Growth mindset	Supportive Therapy (active)	Tar Prev	20–30	20–30	0
Schleider and Weisz (2018)	Growth mindset	Supportive Therapy (active)	Tar Prev	20–30	20–30	39
Schleider et al. (2020)	Growing minds	HEART (active)	Uni Prev	45	NR	17
Schleider et al. (2022)	Project Personality	Sharing Feelings (active)	Tar Prev	NR	NR	0, 13
Shapiro et al. (2022)	IoU focused P‐E	Health focused P‐E (active)	Tar Prev	50	50	0, 1, 4
Shen et al. (2023)	Project RISE	Information only (active)	Tar Prev	20–30	0	0, 2
Short and Schmidt (2020)	FSET Anxiety and Sleep Treatment	Physical Health Education Training (active)	Tar Prev	45	45	1, 4
Steinberg et al. (2024)	Project Think	Project Share ST (active)	Uni Prev	30	30	0, 4, 10, 28

CAST, computerised anxiety sensitivity treatment; CBM‐I, cognitive bias modification; CBT, cognitive behavioural therapy; IoU, intolerance of uncertainty; MI, motivational interviewing; NR, not reported; P‐E, psychoeducation; Tar Prev, targeted prevention; Tar Tx, targeted treatment; Uni Prev, universal prevention.

Fourteen studies were coded as being aimed at targeted prevention (they recruited a targeted sample), five at targeted treatment and six at universal prevention (whereby everyone was eligible to take part). The SSIs implemented a range of approaches from motivational interviewing, psychoeducation, to cognitive bias modification. In terms of control conditions, this varied from passive control (i.e. “waitlist/no intervention controls” and “care as usual”) (four studies), to active controls (e.g. with placebos that matched the SSI in length) (21 studies). Most studies were conducted online or on computers (either in the laboratory, at home or school), with only one reporting to be paper based. Follow‐up length also varied among studies, from immediately posttreatment to 12 months.

### Risk of bias ratings

Several studies were considered to be at high risk of bias (see Figure 2), primarily resulting from biases due to missing outcome data over the course of clinical trials, lack of robust missing data management, and the potential violation of the Missing at Random assumption. Most of the studies did not preregister their trials. One study reported its control and intervention groups to have significantly different ages. Eleven studies were reported to have less than 95% data at follow‐up; 17 of these studies handled missing data using the Intention‐to‐Treat (ITT) approach. It was unclear in three studies whether participants were blinded to the experimental condition.

**Figure 2:**
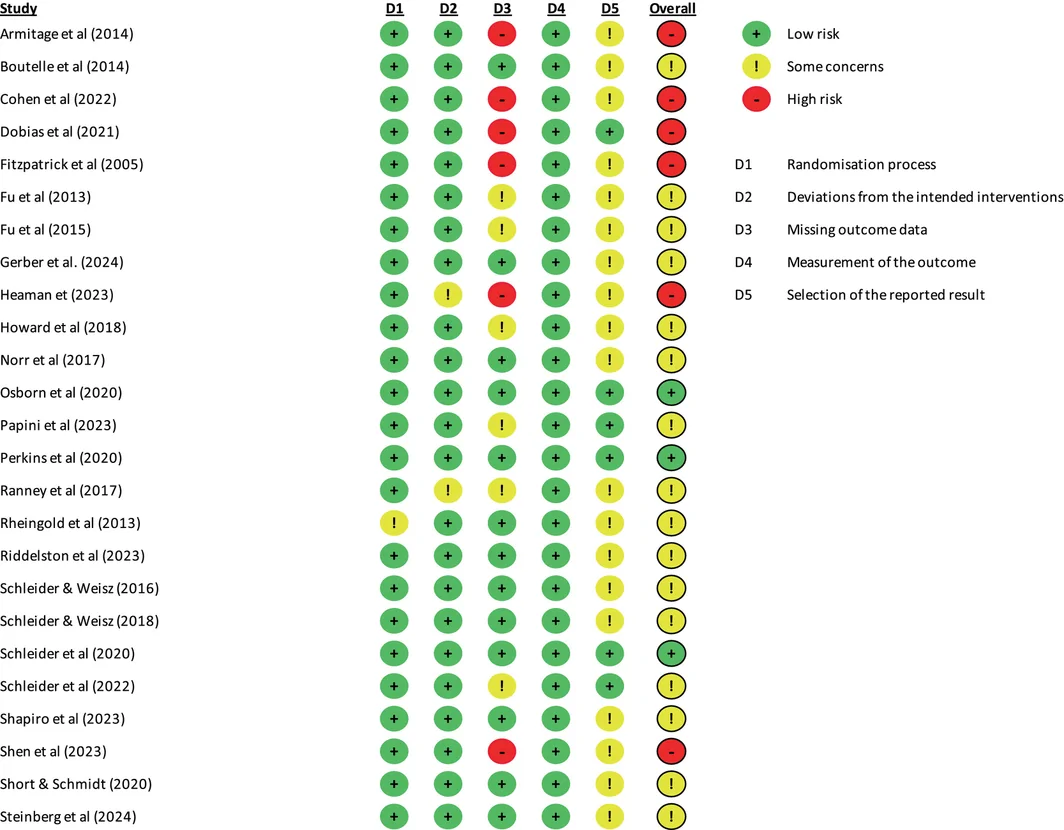
Risk of bias ratings for included studies (*k* = 25)

### Meta‐analysis of intervention effects on anxiety and depressive symptoms

The weighted mean posttreatment Hedges' *g* for the 11 studies that reported an anxiety‐related outcome was −0.24 (95% CI [−0.41, −0.07]) (see Figure 3). The prediction interval ranged from −0.67 to 0.19, meaning that effect sizes in future studies could range from positive benefit to no effect. In the analysis for the anxiety‐related outcomes there was significant substantial heterogeneity (*Q*(10) = 22.88, *p* = .01, *I* ^2^ = 62.68%). A meta‐analysis of 17 studies revealed a small and significant effect of self‐administered SSIs on depressive symptoms (*g* = −0.11, 95% CI [−0.22, −0.02]), with significant substantial heterogeneity (*Q*(16) = 35.95, *p* = .002, *I* ^2^ = 53.86%). Prediction intervals ranged from −0.42 to 0.19, meaning effect sizes in future studies could range from positive benefit to no effect.

**Figure 3:**
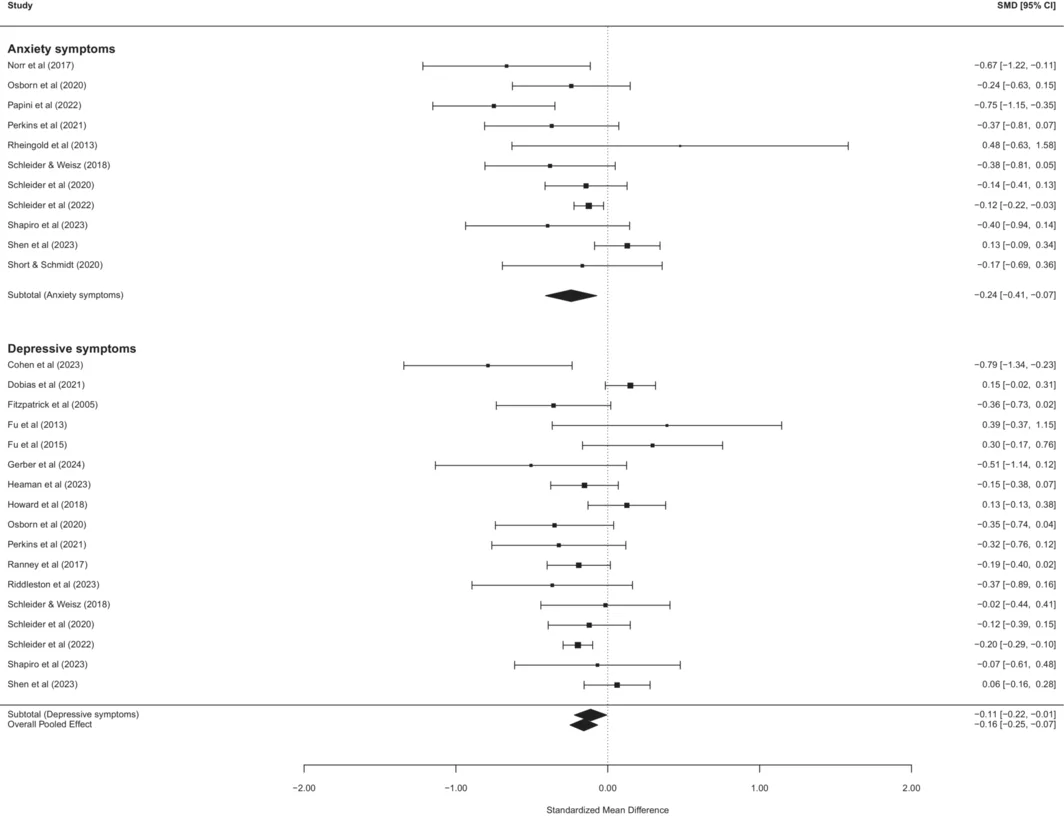
Forest plot showing the pooled effect sizes of self‐administered single‐session interventions on anxiety and depressive symptoms in young people

### Publication bias

Asymmetry was observed when inspecting funnel plots for studies targeting anxiety symptoms (Figure S1). Egger's test did not indicate evidence of publication bias (*z* = −0.96, *p* = .336). However, with only 11 studies, this test has limited power to detect asymmetry, and publication bias cannot be ruled out. Visual inspections of funnel plots (Figure S2) indicated evidence of symmetry for studies targeting depressive symptoms, and this was supported by the results of the Egger's regression test for funnel plot asymmetry, which was nonsignificant (*z* = −1.00, *p* = .318), indicating no evidence of publication bias.

### Sensitivity analysis

Leave‐one‐out sensitivity analyses were conducted. For anxiety symptoms, removing studies by Rheingold, Danielson, Davidson, Self‐Brown, and Resnick (2013) or Shen et al. (2023) resulted in a larger and more statistically significant pooled effect size, suggesting these studies may dampen the overall effect (Figure S3). Heterogeneity decreased slightly upon their removal. For depressive symptoms, removing the study by Dobias, Schleider, Jans, and Fox (2021) resulted in a larger and more statistically significant pooled effect size, alongside a substantial reduction in heterogeneity (*I* ^2^ = 31.98%) (Figure S4). Removing the studies by Cohen, Dobias, Morris, and Schleider (2023) led to a weaker effect.

### Narrative synthesis

#### Substance use

The study by Armitage, Rowe, Arden, and Harris (2014), rating as having ‘High Risk’ of bias, evaluated the effect of their self‐affirming implementation intention SSI on adolescent alcohol use. Compared with a distractor task control condition, their SSI produced a significant decrease in adolescent alcohol consumption at two‐month follow‐up, *F*(1, 64) = 4.62, *p* = .03 (*g =* 0.52, 95% CI [0.03, 1.01]).

#### Psychological flexibility

Perkins, Bowers, Cassidy, Meiser‐Stedman, and Pass (2021) evaluated a mindset intervention that incorporated third‐wave cognitive behavioural constructs to improve adolescent psychological flexibility. Compared with a waitlist control, the intervention showed a moderate effect on psychological flexibility immediately postintervention (*g* = −0.67, 95% CI [−1.12, 0.22]), with the study rated as having a ‘Low Risk’ of bias.

#### Mental well‐being

Osborn et al. (2020) investigated the effects of a computerised intervention called Shamiri‐Digital intervention on adolescents, testing whether teaching growth mindset, gratitude, and value affirmation influences general mental well‐being. Rated as having a ‘Low Risk’ of bias, their study reported a nonsignificant effect on overall mental well‐being at 2‐week follow‐up (*g* = −0.04, 95% CI [−0.54, 0.46]) compared with an active study‐skills control.

#### Eating behaviours

Boutelle, Kuckertz, Carlson, and Amir (2014) tested the Attention Modification Programme (AMP) designed to direct attention away from food cues. With ‘Some Concerns’ regarding risk of bias, this study reported significant treatment effects for overeating, specifically regarding the percentage of daily caloric needs consumed (*g* = −0.97, 95% CI [−1.76, −0.18]) and total calories consumed (*g* = −0.93, 95% CI [−1.72, −0.15]) when comparing to an attention control condition. In contrast to overeating findings, the intervention did not produce a significant group‐by‐time interaction for change in food craving (*g* = −0.50, 95% CI [−0.22, 1.22]).

#### Subgroup analyses and moderator analyses

In the preregistration, we planned to explore whether treatment effects varied based on intervention delivery methods, mental health outcomes, demographic factors, and intervention type. Several post hoc decisions were made during the data analysis stage (See Table S1 for details). First, moderator analysis on intervention delivery method was not conducted, as almost all studies used online delivery, limiting meaningful comparison between different delivery methods. Second, we reported separate meta‐analysis results for studies focusing on anxiety symptoms or depressive symptoms, as these were the most assessed primary outcomes. A moderator analysis was performed to understand if the pooled effect sizes differed between studies assessing anxiety symptoms and those assessed depressive symptoms. The between‐group difference was not statistically significant (*Q*_M_ (1) = 1.43, *p* = .232), indicating that the intervention effect did not significantly differ as a function of outcome type. Studies measuring anxiety symptoms yielded a pooled effect of *g* = −0.24 (95% CI [−0.41, −0.07]), while studies measuring depressive symptoms yielded a pooled effect of *g* = −0.11 (95% CI [−0.22, −0.01]). Third, regarding demographic factors, we considered analysing the impact of participant age group and study country, but the large number of categories with few studies in each made meta‐analysis impractical. For example, moderator analysis by age group was not conducted because the wide variation in age ranges (e.g. late adolescence to young adulthood, early to late adolescence, childhood to early adolescence) would result in too many fragmented and partially overlapping categories. Fourth, moderator analysis on intervention type was not performed due to fewer than four studies in each subgroup. Pooled effect sizes for each intervention type and control type, stratified by anxiety or depressive symptoms, are presented in Table S1. Finally, we conducted moderator analyses to examine whether intervention effect sizes were moderated by SSI duration and follow‐up period. Neither variable showed a statistically significant association with intervention effects (see Table S2).

## Discussion

The aim of this study was to systematically review and synthesise evidence of the efficacy of self‐administered SSIs on mental health outcomes in young people. Meta‐analyses of 19 included studies showed that there was a significant small effect for both anxiety and depressive symptoms, with a significant degree of heterogeneity and a wide prediction interval in each meta‐analysis. About 24% of studies were deemed to be having a ‘High Risk’ of bias, due to high levels of dropout across the intervention period and differences in how missing data were handled. These patterns may suggest potential practical challenges during implementation.

A high level of heterogeneity was observed across studies. Several factors may explain this variability, including differences in treatment type, target problem, population characteristics, and the theoretical underpinnings of the SSIs. However, the small number of included studies limited our ability to test these potential moderators. The wide prediction intervals suggest that effect sizes in future studies may vary considerably, and significant findings may not always replicate, possibly when studies employ different ways to incentivise participation and support engagement at follow‐up.

The magnitude of the pooled effect size across all the studies (*g* = 0.16) is smaller than the one reported in an earlier meta‐analysis (*g* = 0.21) based on 11 studies (Schleider & Weisz, 2017). Both reviews consistently suggest that time‐limited self‐administered SSIs can have a positive impact on children and adolescents' mental health outcomes, with greater effects on anxiety than depressive symptoms. In contrast to Schleider and Weisz (2017), who reported a nonsignificant effect for SSIs on depressive symptoms, the present review provides new evidence of a significant pooled effect for self‐administered SSIs on depressive symptoms (*g* = −0.11). This may be due to the inclusion of more studies (*k* = 11) with increased statistical power. Nevertheless, the lower bound of the confidence interval indicates that the effect on depressive symptoms could be minimal. This aligns with Weisz et al.'s (2023) observation of the ‘Internalising Paradox’, whereby anxiety and depression show divergent responses to psychotherapy.

Moderator analyses conducted in this review indicated that there are no statistically significant differences in pooled effect sizes between studies measuring anxiety‐related outcomes and those on depressive‐related outcomes. In addition, for studies assessing depressive symptom outcomes, there are no significant differences between studies employing active versus passive control. Meta‐regression analyses indicated that the length of SSI delivery and follow‐up period did not significantly predict intervention effects. While understanding moderator effects has potential implications for refining the design and implementation of self‐administered SSIs, this has proven challenging to conduct due to the small number of studies within subgroups. This limited sample size prevented us from conducting subgroup analyses on intervention delivery method (online versus in‐person), participant age group, and study country.

### Strengths and limitations

This review has several strengths. One strength of this review lies in its timeliness. The field has seen a rapid increase in research investment and published trials on online self‐administered SSIs, and an updated synthesis is needed. Additionally, by examining effects across specific symptom domains rather than treating youth mental health as a unitary construct, the review offers a more nuanced understanding of how SSIs affect anxiety and depressive symptoms. Several limitations are noted. First, most studies included in this review were conducted in either Western countries or high‐income countries. A small number were conducted in non‐western countries, such as China and Kenya. Only one of the studies was conducted in a lower‐middle income country like Kenya. Future research should aim to address this gap by exploring the feasibility and effectiveness of self‐administered SSIs in non‐western, lower‐income countries. Second, unpublished, non‐peer‐reviewed studies were not included in this review, although this limitation is mitigated by the absence of evidence for publication bias in our sensitivity analysis. Third, moderator analysis could not be performed on certain moderators that have many categories yet too few studies in each category. Fourth, applying the same search terms across multiple databases may carry the risk of overlooking relevant studies. However, this limitation was mitigated by conducting citation search and updated search. Fifth, RoB‐2 Domain 3 can yield positive ratings when the attrition is low, even in the absence of ITT. The included studies were brief, self‐administered SSIs with relatively low attrition at the first available posttreatment time point. Having ‘Low Risk’ RoB‐2 Domain 3 ratings should not be taken as evidence that missing data were handled using best practice analytic methods. Finally, a systematic evaluation of engagement and attrition across included trials was beyond the scope of the current review. Future reviews examining implementation outcomes would be a valuable complement to the present efficacy‐focused findings.

### Research implications

Our quality review identified engagement challenges across studies, including varying randomisation adherence, attrition postrandomisation, incomplete postassessment outcomes, and significant follow‐up loss. These issues not only limit the generalisability of findings and potentially introduce selection bias but also raise concerns about uptake of interventions in real‐world settings. Further systematic review may explore whether specific design elements are associated with these engagement metrics. Understanding and mitigating attrition is crucial for improving internal validity and enhancing the potential for successful adoption. Future empirical research should consider altering intervention design factors that may boost treatment efficacy, such as follow‐up initiatives, anonymous participation, contact with a real person, participatory co‐production, and theoretical modalities. In addition, SSIs may benefit from incorporating machine learning techniques, large language models, and chatbots to reduce barriers for users to access mental health support (Loades & Schleider, 2023). It would be important for future research to investigate factors that promote lasting effects of SSIs. For example, supportive cues have been shown to enhance the longevity of SSI effects and effects can persist at 9 months postintervention (Hecht et al., 2023; Schleider & Weisz, 2017). There is limited availability of evidence for the effects of self‐administered SSIs on concepts other than anxiety and depressive symptoms. Given the multifaceted nature of mental health, more research is needed to assess other aspects of mental health indicators, such as self‐esteem, stress, compassion, and psychological flexibility (Becker, Chorpita, & Daleiden, 2011). Given the lack of studies that recruited participants from non‐western, low‐income countries, future research could aim to explore how to adapt SSIs to make them acceptable to individuals in non‐western countries. Research could also explore co‐production. For example, in this review, Riddleston, Bangura, Gibson, Qualter, and Lau (2023) co‐created their cognitive modification SSI with young people with lived experience, to ensure they targeted the concept of loneliness, and suggested that this improved acceptability and efficacy.

### Clinical implications

In terms of clinical applications, this review highlights the potential and benefits SSIs can bring to improve young people's mental health. With timely access, we might be more likely to see improvements in positive indicators of mental health. Furthermore, given the utility of SSIs and their ability to overcome access barriers, they could potentially reduce service burden as there is no need for clinicians to facilitate them, and no expectation of a second visit since the ‘treatment dose’ is achieved in the single session, unlike conventional digital interventions which have been subject to drop out (Loades & Schleider, 2023). Although certain forms of digital guided self‐help have now been recognised by the National Institute of Clinical Excellence (NICE) Early Value Assessment as a recommended tool for young people (NICE, 2023), further research and service evaluations are needed to assess whether SSIs could serve as a potential form of early intervention, subject to real‐world evaluation and the development of high certainty evidence. This review also demonstrates that SSIs could benefit young people who are often less likely to access traditional mental health support (e.g. Shen et al., 2023). Furthermore, given that most studies were conducted online, this demonstrates their accessibility given 97% of young people have access to the internet (Ofcom, 2023) and aligns with the NHS long‐term plans of transforming mental health care to integrate the digital world (Health Education England, 2019), suggesting they could have a role in future service delivery.

## Conclusions

As mental health care for young people continues to evolve, self‐administered SSIs are gaining traction. This meta‐analytic review found small but promising effects in reducing anxiety and depressive symptoms in young people, highlighting their value in promoting mental well‐being at scale. The potential real‐world impact of self‐administered SSIs will depend not only on their content but also on improving both initiation and sustained engagement.

## Ethical considerations

This study is a systematic review of published literature and did not involve the collection of primary data from human participants. Informed consent/assent was therefore not applicable, and formal ethical approval from a Research Ethics Committee or Institutional Review Board was not required.

## Trial registration

This random‐effects meta‐analytic review was preregistered on PROSPERO. Registration number: CRD42023457030, registered 19 September 2023, available at: https://www.crd.york.ac.uk/PROSPERO/view/CRD42023457030.

**Key points**

**What's known?**

Young people face significant barriers to accessing mental health support. Self‐administered Single‐Session Interventions (SSIs) have arisen as one potential solution.

**What's new?**

This is the first meta‐analytic review focused on the efficacy of self‐administered SSIs for youth mental health, with results showing small and significant effects in reducing anxiety and depressive symptoms.

**What's relevant?**

Self‐administered SSIs are effective, scalable, and accessible, making them potentially suitable for addressing youth mental health needs.

## Supporting information


**Appendix S1.** PRISMA checklist.
**Figure S1**. Funnel plot for studies assessing anxiety symptom outcomes, with trim‐and‐fill method applied (*k* = 11).
**Figure S2**. Funnel plot for studies assessing depressive symptom outcomes (*k* = 17).
**Figure S3**. Leave‐one‐out sensitivity analysis for studies assessing anxiety symptom outcomes (*k* = 11).
**Figure S4**. Leave‐one‐out sensitivity analysis for studies assessing depressive symptoms (*k* = 17).
**Table S1**. Subgroup analyses and moderator analyses.
**Table S2**. Meta‐regression results examining the effect of moderator variables on pooled effect sizes.

## Data Availability

This article is a meta‐analysis and does not contain any original data. All data analysed in this study were extracted from previously published studies, which are cited in the reference list.

## References

[jcpp70192-bib-0001] Arain, M. , Haque, M. , Johal, L. , Mathur, P. , Nel, W. , Rais, A. , … & Sharma, S. (2013). Maturation of the adolescent brain. Neuropsychiatric Disease and Treatment, 9, 449–461. 10.2147/NDT.S39776 PMC3621648 23579318

[jcpp70192-bib-0002] Armitage, C.J. , Rowe, R. , Arden, M.A. , & Harris, P.R. (2014). A brief psychological intervention that reduces adolescent alcohol consumption. Journal of Consulting and Clinical Psychology, 82, 546–550. 10.1037/a0035802 24491079

[jcpp70192-bib-0003] Becker, K.D. , Chorpita, B.F. , & Daleiden, E.L. (2011). Improvement in symptoms versus functioning: How do our best treatments measure up? Administration and Policy in Mental Health and Mental Health Services Research, 38, 440–458. 10.1007/s10488-010-0332-x 21207129

[jcpp70192-bib-0004] Borenstein, M. (2023). How to understand and report heterogeneity in a meta‐analysis: The difference between I‐squared and prediction intervals. Integrative Medicine Research, 12, 101014. 10.1016/j.imr.2023.101014 PMC11208730 38938910

[jcpp70192-bib-0005] Boutelle, K.N. , Kuckertz, J.M. , Carlson, J. , & Amir, N. (2014). A pilot study evaluating a one‐session attention modification training to decrease overeating in obese children. Appetite, 76, 180–185. 10.1016/j.appet.2014.01.075 PMC3989523 24512975

[jcpp70192-bib-0006] Cohen, J. (2013). Statistical power analysis for the behavioral sciences (2nd ed.). Routledge. 10.4324/9780203771587.

[jcpp70192-bib-0007] Cohen, K. , Dobias, M. , Morris, R.R. , & Schleider, J.L. (2023). Improving uptake of mental health crisis resources: Randomized test of a single‐session intervention embedded in social media. Journal of Behavioral and Cognitive Therapy, 33, 24–34.

[jcpp70192-bib-0008] Davies, G. (2023). Progress in improving mental health services in England. National Audit Office. Available from: https://www.nao.org.uk/wpcontent/uploads/2023/02/Progress‐in‐improving‐mental‐health‐services‐CS.pdf [last accessed 14 January 2024].

[jcpp70192-bib-0009] Dettori, J.R. , Norvell, D.C. , & Chapman, J.R. (2022). Fixed‐effect vs random‐effects models for meta‐analysis: 3 points to consider. Global Spine Journal, 12, 1624–1626. 10.1177/21925682221110527 PMC9393987 35723546

[jcpp70192-bib-0010] Dobias, M. , Schleider, J.L. , Jans, L. , & Fox, K. (2021). An online, single‐session intervention for adolescent self‐injurious thoughts and behaviors: Results from a randomized trial. Behaviour Research and Therapy, 147, 103983. 10.1016/j.brat.2021.103983 34688102

[jcpp70192-bib-0012] Duval, S. , & Tweedie, R.L. (2000). Trim and fill: A simple funnel‐plot–based method of testing and adjusting for publication bias in meta‐analysis. Biometrics, 56, 455–463. 10.1111/j.0006-341x.2000.00455.x 10877304

[jcpp70192-bib-0013] Edbrooke‐Childs, J. , & Deighton, J. (2020). Problem severity and waiting times for young people accessing mental health services. British Journal of Psychiatry Open, 6, e118. 10.1192/bjo.2020.103 PMC7576668 33040766

[jcpp70192-bib-0014] Fegert, J.M. , Vitiello, B. , Plener, P.L. , & Clemens, V. (2020). Challenges and burden of the coronavirus 2019 (COVID‐19) pandemic for child and adolescent mental health: A narrative review to highlight clinical and research needs in the acute phase and the long return to normality. Child and Adolescent Psychiatry and Mental Health, 14, 20. 10.1186/s13034-020-00329-3 PMC7216870 32419840

[jcpp70192-bib-0015] Fitzpatrick, K.K. , Witte, T.K. , & Schmidt, N.B. (2005). Randomized controlled trial of a brief problem‐orientation intervention for suicidal ideation. Behavior Therapy, 36, 323–333.

[jcpp70192-bib-0016] Fu, X. , Du, Y. , Au, S. , & Lau, J.Y.F. (2013). Reducing negative interpretations in adolescents with anxiety disorders: A preliminary study investigating the effects of a single session of cognitive bias modification training. Developmental Cognitive Neuroscience, 4, 29–37. 10.1016/j.dcn.2012.11.003 PMC6987770 23219491

[jcpp70192-bib-0017] Fu, X. , Du, Y. , Au, S. , & Lau, J.Y.F. (2015). Single‐session cognitive bias modification of interpretations training in high‐anxious adolescents. Journal of Cognitive Psychotherapy, 29, 253–272. 10.1891/0889-8391.29.3.253 32755951

[jcpp70192-bib-0018] Gerber, A.H. , Nahmias, A. , Schleider, J.L. , & Lerner, M.D. (2024). Results from a pilot randomized controlled trial of a single‐session growth‐mindset intervention for internalizing symptoms in autistic youth. Journal of Autism and Developmental Disorders, 55, 3050–3064.10.1007/s10803-024-06341-238833031

[jcpp70192-bib-0019] Gulliver, A. , Griffiths, K.M. , & Christensen, H. (2010). Perceived barriers and facilitators to mental health help‐seeking in young people: A systematic review. BMC Psychiatry, 10, 113. 10.1186/1471-244X-10-113 PMC3022639 21192795

[jcpp70192-bib-0020] Health Education England . (2019). The digital future of mental healthcare and its workforce: A report on mental health stakeholder engagement to inform the Topol Review. NHS England. Available from: https://topol.hee.nhs.uk/wp‐content/uploads/HEE‐Topol‐Review‐Mental‐health‐paper.pdf [last accessed 29 February 2024].

[jcpp70192-bib-0021] Heaman, J.A.L. , Cherry, K.M. , McMurtry, C.M. , Giguère, B. , & Lumley, M.N. (2023). Changing minds: An RCT of a growth mindset intervention on depressive symptoms and well‐being in adolescent girls and boys. International Journal of Applied Positive Psychology, 9, 381–409.

[jcpp70192-bib-0022] Hecht, C.A. , Gosling, S.D. , Bryan, C.J. , Jamieson, J.P. , Murray, J.S. , & Yeager, D.S. (2023). When do the effects of single‐session interventions persist? Testing the mindset + supportive context hypothesis in a longitudinal randomized trial. JCPP Advances, 3, e12191. 10.1002/jcv2.12191 PMC10694537 38054060

[jcpp70192-bib-0025] Howard, K.A. , Griffiths, K.M. , McKetin, R. , & Ma, J. (2018). Can a brief biologically based psychoeducational intervention reduce stigma and increase help‐seeking intentions for depression in young people? A randomised controlled trial. Journal of Child & Adolescent Mental Health, 30, 27–39. 10.2989/17280583.2018.1467323 29764293

[jcpp70192-bib-0026] Lehtimaki, S. , Martic, J. , Wahl, B. , Foster, K.T. , & Schwalbe, N. (2021). Evidence on digital mental health interventions for adolescents and young people: Systematic overview. JMIR Mental Health, 8, e25847. 10.2196/25847 PMC8120421 33913817

[jcpp70192-bib-0027] Loades, M. , & Schleider, J.L. (2023). Technology matters: Online, self‐help single session interventions could expand current provision, improving early access to help for young people with depression symptoms, including minority groups. Child and Adolescent Mental Health, 28, 559–561. 10.1111/camh.12659 PMC10592457 37415054

[jcpp70192-bib-0028] National Institute for Health and Care Excellence . (2023). Guided self‐help digital cognitive behavioural therapy for children and young people with mild to moderate symptoms of anxiety or low mood: early value assessment. Available from: https://www.nice.org.uk/guidance/hte3.

[jcpp70192-bib-0029] Norr, A.M. , Gibby, B.A. , & Schmidt, N.B. (2017). Is computerized psychoeducation sufficient to reduce anxiety sensitivity in an at‐risk sample?: A randomized trial. Journal of Affective Disorders, 212, 48–55. 10.1016/j.jad.2017.01.032 28142083

[jcpp70192-bib-0030] Ofcom . (2023). Children and parents: Media use and attitudes. Available from: https://www.ofcom.org.uk/__data/assets/pdf_file/0027/255852/childrens‐media‐use‐and‐attitudes‐report‐2023.pdf [last accessed March 3, 2024].

[jcpp70192-bib-0031] Osborn, T.L. , Rodríguez, M. , Wasil, A.R. , Venturo‐Conerly, K.E. , Gan, J. , Alemu, R. , … & Shingleton, R. (2020). Single‐session digital intervention for adolescent depression, anxiety, and well‐being: Outcomes of a randomized controlled trial with Kenyan adolescents. Journal of Consulting and Clinical Psychology, 88, 657–668. 10.1037/ccp0000505 32391709

[jcpp70192-bib-0032] Ouzzani, M. , Hammady, H.M. , Fedorowicz, Z. , & Elmagarmid, A.K. (2016). Rayyan—A web and mobile app for systematic reviews. Systematic Reviews, 5, 210. 10.1186/s13643-016-0384-4 PMC5139140 27919275

[jcpp70192-bib-0034] Papini, S. , Jacquart, J. , Zaizar, E.D. , Telch, M.J. , & Smits, J.A.J. (2023). Targeting anxiety sensitivity with evidence‐based psychoeducation: A randomized waitlist‐controlled trial of a brief standalone digital intervention. Cognitive and Behavioral Practice, 30, 678–691.

[jcpp70192-bib-0035] Perkins, A. , Bowers, G. , Cassidy, J.D. , Meiser‐Stedman, R. , & Pass, L. (2021). An enhanced psychological mindset intervention to promote adolescent wellbeing within educational settings: A feasibility randomized controlled trial. Journal of Clinical Psychology, 77, 946–967. 10.1002/jclp.23104 33450060

[jcpp70192-bib-0036] R Core Team . (2025). R: A language and environment for statistical computing. Vienna, Austria: R Foundation for Statistical Computing. Available from: https://www.R‐project.org/.

[jcpp70192-bib-0037] Racine, N. , McArthur, B.A. , Cooke, J.E. , Eirich, R. , Zhu, J. , & Madigan, S. (2021). Global prevalence of depressive and anxiety symptoms in children and adolescents during COVID‐19. JAMA Pediatrics, 175, 1142–1150. 10.1001/jamapediatrics.2021.2482 PMC8353576 34369987

[jcpp70192-bib-0038] Radež, J. , Reardon, T. , Creswell, C. , Lawrence, P.J. , Evdoka‐Burton, G. , & Waite, P. (2020). Why do children and adolescents (not) seek and access professional help for their mental health problems? A systematic review of quantitative and qualitative studies. European Child & Adolescent Psychiatry, 30, 183–211. 10.1007/s00787-019-01469-4 PMC7932953 31965309

[jcpp70192-bib-0039] Ranney, M.L. , Goldstick, J.E. , Eisman, A.B. , Carter, P.M. , Walton, M.A. , & Cunningham, R.M. (2017). Effects of a brief ED‐based alcohol and violence intervention on depressive symptoms. General Hospital Psychiatry, 46, 44–48. 10.1016/j.genhosppsych.2017.01.008 PMC5474937 28622815

[jcpp70192-bib-0040] Rheingold, A.A. , Danielson, C.K. , Davidson, T.M. , Self‐Brown, S. , & Resnick, H.S. (2013). Video intervention for child and caregiver distress related to the child sexual abuse medical examination: A randomized controlled pilot study. Journal of Child and Family Studies, 22, 386–397. 10.1080/10538712.2013.800937 23837655

[jcpp70192-bib-0041] Riddleston, L. , Bangura, E. , Gibson, O. , Qualter, P. , & Lau, J.Y.F. (2023). Developing an interpretation bias modification training task for alleviating loneliness in young people. Behaviour Research and Therapy, 168, 104380. 10.1016/j.brat.2023.104380 37541156

[jcpp70192-bib-0042] Schleider, J.L. , Burnette, J.L. , Widman, L. , Hoyt, C.L. , & Prinstein, M.J. (2020). Randomized trial of a single‐session growth mind‐set intervention for rural adolescents' internalizing and externalizing problems. Journal of Clinical Child and Adolescent Psychology, 49, 660–672. 10.1080/15374416.2019.1622123 PMC6923626 31219698

[jcpp70192-bib-0043] Schleider, J.L. , Dobias, M. , Sung, J. , & Mullarkey, M.C. (2019). Future directions in single‐session youth mental health interventions. Journal of Clinical Child and Adolescent Psychology, 49, 264–278. 10.1080/15374416.2019.1683852 PMC7065925 31799863

[jcpp70192-bib-0044] Schleider, J.L. , Mullarkey, M.C. , Fox, K. , Dobias, M. , Shroff, A. , Hart, E.A. , & Roulston, C.A. (2022). A randomized trial of online single‐session interventions for adolescent depression during COVID‐19. Nature Human Behaviour, 6, 258–268. 10.1038/s41562-021-01235-0 PMC8881339 34887544

[jcpp70192-bib-0045] Schleider, J.L. , & Weisz, J.R. (2018). A single‐session growth mindset intervention for adolescent anxiety and depression: 9‐month outcomes of a randomized trial. Journal of Child Psychology and Psychiatry, 59, 160–170. 10.1111/jcpp.12811 28921523

[jcpp70192-bib-0046] Schleider, J.L. , & Weisz, J.R. (2016). Reducing risk for anxiety and depression in adolescents: Effects of a single‐session intervention teaching that personality can change. Behaviour Research and Therapy, 87, 170–181. 10.1016/j.brat.2016.09.011 PMC5127737 27697671

[jcpp70192-bib-0047] Schleider, J.L. , & Weisz, J.R. (2017). Can less be more? The promise (and perils) of single‐session youth mental health interventions. The Behavior Therapist, 40, 256–261.

[jcpp70192-bib-0048] Schwarzer, G. , Carpenter, J.R. , & Rücker, G. (2015). Meta‐analysis with binary outcomes. In G. Schwarzer , J.R. Carpenter , & G. Rücker (Eds.), Meta‐analysis with R (pp. 55–83). Cham: Springer international publishing.

[jcpp70192-bib-0049] Shapiro, M.O. , Allan, N.P. , Raines, A.M. , & Schmidt, N.B. (2022). A randomized control trial examining the initial efficacy of an intolerance of uncertainty focused psychoeducation intervention. Journal of Psychopathology and Behavioral Assessment, 45, 379–390.

[jcpp70192-bib-0050] Shen, J. , Rubin, A. , Cohen, K.E. , Hart, E.A. , Sung, J.E. , McDanal, R. , … & Schleider, J.L. (2023). Randomized evaluation of an online single‐session intervention for minority stress in LGBTQ+ adolescents. Internet Interventions, 33, 100633. 10.1016/j.invent.2023.100633 PMC10457524 37635950

[jcpp70192-bib-0051] Short, N.A. , & Schmidt, N.B. (2020). Developing and testing a novel, computerized insomnia and anxiety intervention to reduce safety AIDs among an at‐risk student sample: A randomized controlled trial. Behavior Therapy, 51, 149–161. 10.1016/j.beth.2019.05.012 32005332

[jcpp70192-bib-0052] Steinberg, J.S. , Fitzpatrick, O.M. , Khurana, S. , Kim, M.Y. , Mair, P. , Schleider, J.L. , … & Weisz, J.R. (2024). Is there a place for cognitive restructuring in brief, self‐guided interventions? Randomized controlled trial of a single‐session, digital program for adolescents. Journal of Clinical Child and Adolescent Psychology, 1–21, 1–21. 10.1080/15374416.2024.2384026 39120779

[jcpp70192-bib-0053] Sterne, J.A.C. , Savović, J. , Page, M.J. , Elbers, R.G. , Blencowe, N.S. , Boutron, I. , … & Higgins, J.P.T. (2019). RoB 2: A revised tool for assessing risk of bias in randomised trials. BMJ (Clinical Research Ed.), 366, l4898. 10.1136/bmj.l4898 31462531

[jcpp70192-bib-0054] Tunks, A. , Berry, C. , Strauss, C. , Nyikavaranda, P. , & Ford, E. (2023). Patients' perspectives of barriers and facilitators to accessing support through primary care for common mental health problems in England: A systematic review. Journal of Affective Disorders, 338, 329–340. 10.1016/j.jad.2023.06.035 37348656

[jcpp70192-bib-0055] Viechtbauer, W. (2010). Conducting meta‐analyses in R with the metafor package. Journal of Statistical Software, 36, 1–48.

[jcpp70192-bib-0056] Wang, Q. , Zhang, W. , & An, S. (2023). A systematic review and meta‐analysis of internet‐based self‐help interventions for mental health among adolescents and college students. Internet Interventions, 34, 100690. 10.1016/j.invent.2023.100690 PMC10654252 38023965

[jcpp70192-bib-0058] Weisz, J.R. , Fitzpatrick, O.M. , Venturo‐Conerly, K.E. , Sternberg, A. , Steinberg, J.S. , & Ng, M.Y. (2023). Research review: The internalizing paradox – youth anxiety and depression symptoms, psychotherapy outcomes, and implications for research and practice. Journal of Child Psychology and Psychiatry, 64, 1720–1734. 10.1111/jcpp.13820 PMC10667566 37222162

[jcpp70192-bib-0059] World Health Organization: WHO . (2022). Self‐care interventions for health. Available from: https://www.who.int/news‐room/fact‐sheets/detail/self‐care‐health‐interventions#:~:text=Self%2Dcare%20interventions%20promote%20individuals,autonomy%20and%20engagement%20in%20health.

